# Potential Relationship Between Lifestyle Changes and Incidence of Hospital Admissions for Acute Coronary Syndrome During the COVID-19 Lockdown

**DOI:** 10.3389/fcvm.2021.604374

**Published:** 2021-02-11

**Authors:** Grigorios Tsigkas, Eleni-Evangelia Koufou, Konstantinos Katsanos, Panagiotis Patrinos, Athanasios Moulias, Ioannis Miliordos, Georgios Almpanis, Ioannis Christodoulou, Fotini Papanikolaou, Theodora Dimitroula, Andreas Kivetos, Panagiotis Vardas, Periklis Davlouros

**Affiliations:** ^1^Department of Cardiology, Patras University Hospital, Patras, Greece; ^2^Department of Radiology, Patras University Hospital, Patras, Greece; ^3^Department of Cardiology, General Hospital of Agrinio, Agrinio, Greece; ^4^Department of Cardiology, General Hospital of Patras, Patras, Greece; ^5^Department of Cardiology, General Hospital of Pyrgos, Pyrgos, Greece; ^6^Department of Cardiology, University Hospital of Heraklion, Heraklion, Greece

**Keywords:** ACS, COVID-19, lifestyle—related disease, way of life, stabilization of atherosclerotic plaque

## Abstract

**Aims:** To evaluate the impact of lockdown during the COVID-19 pandemic on lifestyle changes of the general population, and on admissions for acute coronary syndrome (ACS).

**Methods and Results:** All ACS admissions during the COVID-19 lockdown (10 March to 4 May, 2020), in 3 municipalities (3 spoke, and 1 hub hospital), in Southwestern Greece (411,576 inhabitants), were prospectively recorded and compared to the equivalent periods during 2018, and 2019. A telephone survey of 1014 participants was conducted to explore the lifestyle habits of citizens aged ≥35-years-old before and during lockdown. The median ACS incidence rate decreased from 19.0 cases per week in 2018 and 21.5 in 2019 down to 13.0 in 2020 (RR: 0.66 during the Covid-19 lockdown; 95%CI: 0.53–0.82; *P* = 0.0002). This was driven by a significant reduction of admissions for Non-ST elevation myocardial infarction (NSTEMI) (RR: 0.68; 95%CI: 0.52–0.88; *P* = 0.0037), mainly in patients with a lower burden of cardiovascular risk factors, as we noticed an inverse association between the reduction of the incidence of ACS during the Covid-19 lockdown period and the number of registered patient risk factors. There was no difference in the rates of STEMI and population-based all-cause mortality across the examined time periods. The telephone survey demonstrated reduction of passive smoking, working hours, alcohol, junk food and salt consumption, and an increase in sleeping hours, mainly in participants with a lower burden of cardiovascular risk factors.

**Conclusions:** A significant decline in ACS admissions during the COVID-19 lockdown was noted, affecting mainly NSTEMI patients with a lower burden of cardiovascular risk factors. This was accompanied by significant lifestyle changes. Thus, it is tempting to speculate that to some extend the latter might be associated with the observed decline in ACS admissions.

## Introduction

The coronavirus disease (Covid-19) pandemic has become a major cause of mortality worldwide. This has led to the adoption of social distancing measures, or even a complete lockdown policy over various timeframes, which have been ordered by many administrations in Europe, and the USA in an effort to restrict virus transmission. The impact of this extremely unique situation on a population's lifestyle habits has not been studied. During the above lockdown periods a decrease in hospital admissions for acute coronary syndrome (ACS) has been observed (1–6). The prevailing explanatory theory on this observation is that patients may have avoided seeking medical help through fear of the pandemic, thus causing a false decrease in the rate of ACS. However, we cannot exclude a real decrease in ACS incidence due to lifestyle changes associated with the enforced quarantine, especially in countries where the cases of COVID-19, and the resulting number of deaths were kept quite low, without significantly stressing of the health system. Greece is such a country, where strict quarantine and major lockdown measures were instituted at the very beginning of the outbreak.

The aim of the present study was (1) to compare the prospectively recorded rates of hospital admissions for ACS during the lockdown time interval (Covid-19 era, 10 March to 4 May 2020) with those during the same interval in the years 2018 and 2019 (pre-Covid-19 era, over a large network consisting of 1 hub, and 3 spoke hospitals in southwestern Greece (411,576 inhabitants); and, (2) to determine via a telephone interview survey any changes in citizens' basic lifestyle habits (exercise, sleep, smoking, diet, etc.), during and before the lockdown, that may have contributed to cardiovascular risk modification and a potential reduction in ACS incidence.

## Methods

The first CoVid-19 case in Greece was reported on 20 February 2020; strict social distancing was instituted by 10 March 2020; on 11 March 2020 the World Health Organization declared the outbreak a pandemic; and lockdown was imposed in Greece on 13 March 2020 lasting until 4 May 2020. This research was confined to southwestern Greece, and included 3 large municipalities with 411,576 inhabitants according to the last national census of 2011. Our hospital is the only hospital with a hemodynamic laboratory in southwestern Greece, and offers a primary percutaneous intervention (PCI) service on a 24/7 basis, being the hub hospital for 3 large general district hospitals (spoke hospitals). We prospectively recorded admissions for ACS in all hospitals during the period of strict social distancing and lockdown in Greece (10 March to 4 May, 2020), and searched all hospitals' databases for admissions with ACS [ST-elevation myocardial infarction (STEMI) and Non-ST-elevation MI (NSTEMI)], during the corresponding period in 2018 and 2019. Total population all-cause death rate was collected by the three large municipalities for the corresponding period over the last 3 years (2018–2020).

A telephone survey was conducted between 13 and 30 April, 2020, by a certified to implement a Quality Management System company (DATA RC SA, ISO 9001:2015 & Information Security ISO 27001:2013), to explore the lifestyle habits of citizens during and before quarantine. The survey sample was designed to represent the general population of the region of southwestern Greece aged ≥35 years old, in terms of geographical criteria (3 regional units). Data were collected via telephone interviews using CATI (Computer Assisted Telephone Interviewing) technology, conducted by experienced interviewers who read and completed the survey questionnaire remotely. Each respondent was asked to give his/ hers explicit consent in order to participate in the survey, in accordance with General Data Protection Regulation (GDPR) rules. Additionally, 20% of the questionnaires were cross-checked by the field manager who monitored the call. The duration of the interview was about 8 min and it was based on a strictly structured questionnaire that included a brief medical history, lifestyle data (smoking, alcohol consumption, hours of sleep, and work, type of diet, exercise), as well as self-evaluation of anxiety related to the pandemic, and depressive feelings during and before the application of lockdown.

### Statistical Analysis

Patient characteristics were extracted from electronic medical records and defined according to international guidelines and standards of good practice. We calculated weekly counts of ACS admissions and ensuing urgent coronary revascularization across the regional hub-and-spoke referral network. We also looked separately into weekly counts of the types of ascertained ACS sub-diagnosis (NSTEMI vs. STEMI) and type of revascularization [percutaneous coronary intervention (PCI) vs. coronary artery bypass grafting (CABG)]. Population-based mortality rates were obtained from local municipal archives. Counts of events were stratified by year and by pre-CoVid-19 (years, 2018 and 2019) and Covid-19 period (year 2020). The same time interval (10 March to 04 May) was examined in each year to account for seasonal variations. Missing data (<0.5%) were filled by multivariate imputation with chained equations (Missing-At-Random principle). To compare patient covariates during the 3 years (2018, 2019, and 2020), one-way ANOVA was used for continuous variables and the chi square test was used for explanatory categorical variables. Stratified analyses were also performed for various patient risk factors as reported in the results section and respective tables. Generalized linear models were applied to investigate the associations between response and explanatory variables. A Poisson log-likelihood function was fitted to regress weekly counts of ACS admissions. Overdispersion was excluded by comparing residual deviance to degrees of freedom. Multicollinearity was excluded by calculation of variance inflation factors. We generated plots of the weekly incidence of ACS and studied subgroups, as well as of different patient strata to demonstrate the observed effect of the pandemic lockdown. All variables recorded in the telephone survey were classified as categorical and tested with the chi-square test. Logistic regression models were fitted to regress lifestyle habit changes collected by the telephone survey. To address the familywise error rate arising from multiple testing, we generously adjusted the level of type I error to α = 0.1% by the stringent Bonferroni method (i.e., statistical significance was assumed for *p* < 0.001). All statistical analyses were performed in the R language environment (version 3.6.3).

## Results

**Figure 1:**
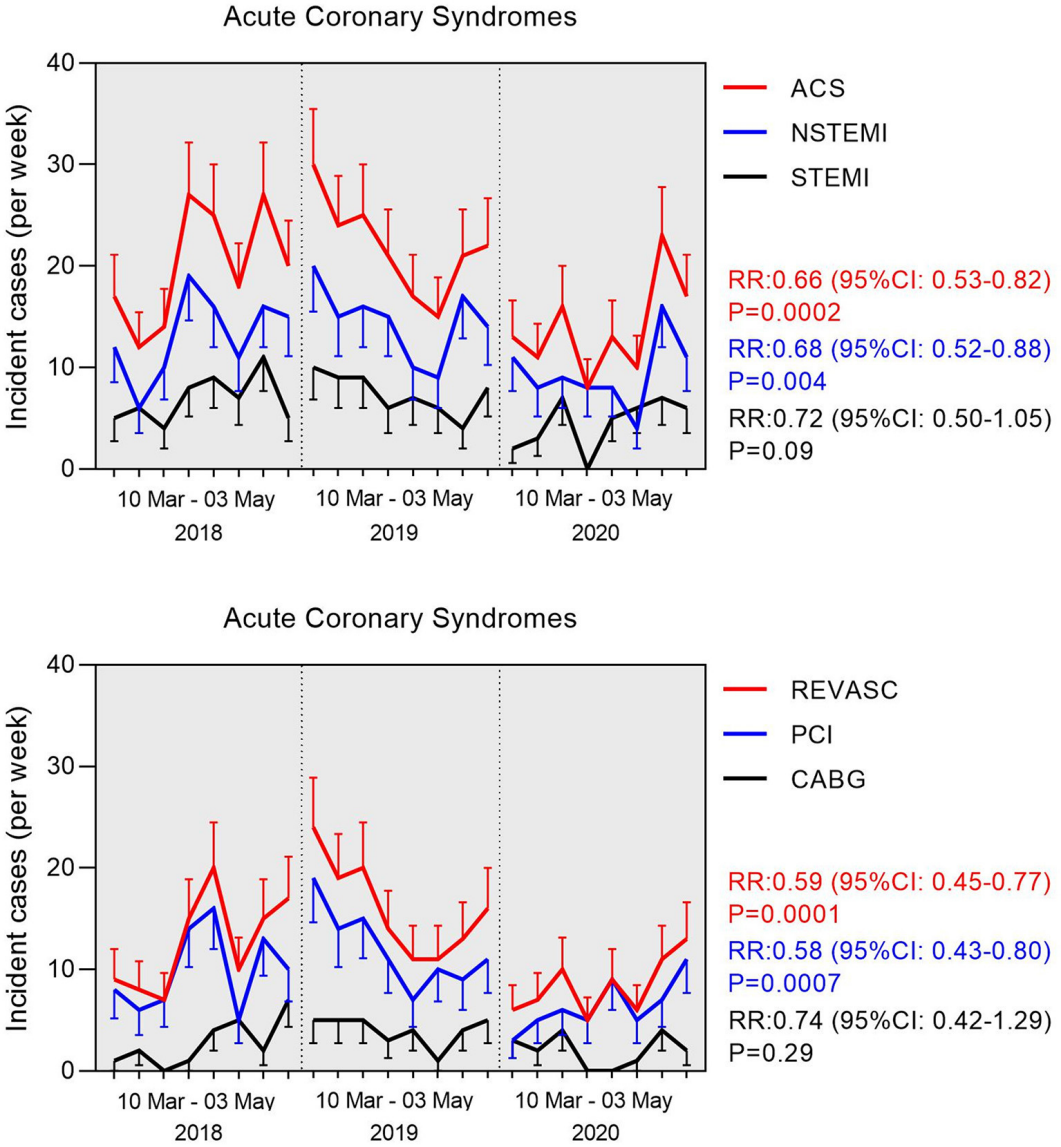
Weekly counts of incident cases of acute coronary syndrome (ACS) between 10 March and 04 May (period of 8 weeks) during 2018, 2019, and 2020. **(Top)** Overall ACS incidence stratified by type of ACS (STEMI vs. NSTEMI). **(Bottom)** Total counts of ACS cases that underwent revascularization (REVASC) stratified by mode (PCI vs. CABG). Relative risks (RR) were calculated by Poisson regression adjusting for Covid-19 epidemic.

**Figure 2:**
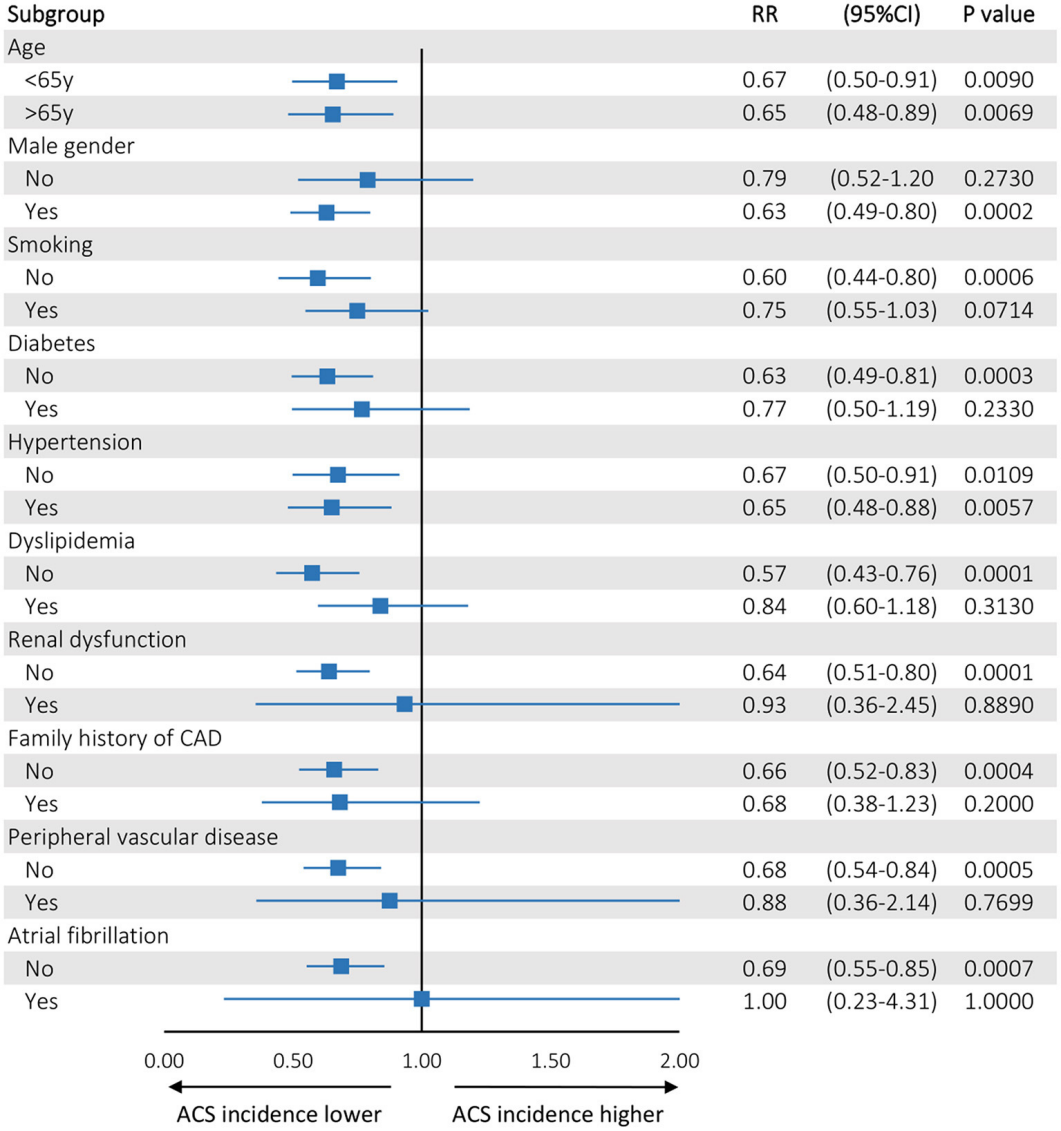
Forest plot showing the results (Risk Ratios) of regression analysis of the weekly incidence of ACS across different strata of registered patient characteristics. Age was dichotomized at its median value (65 years)—rest of the remaining categorical explanatory variables were stratified by factor (GLM with a Poisson log-likelihood function). Statistical significance was assumed for *P* < 0.001 to account for multiple testing.

**Figure 3:**
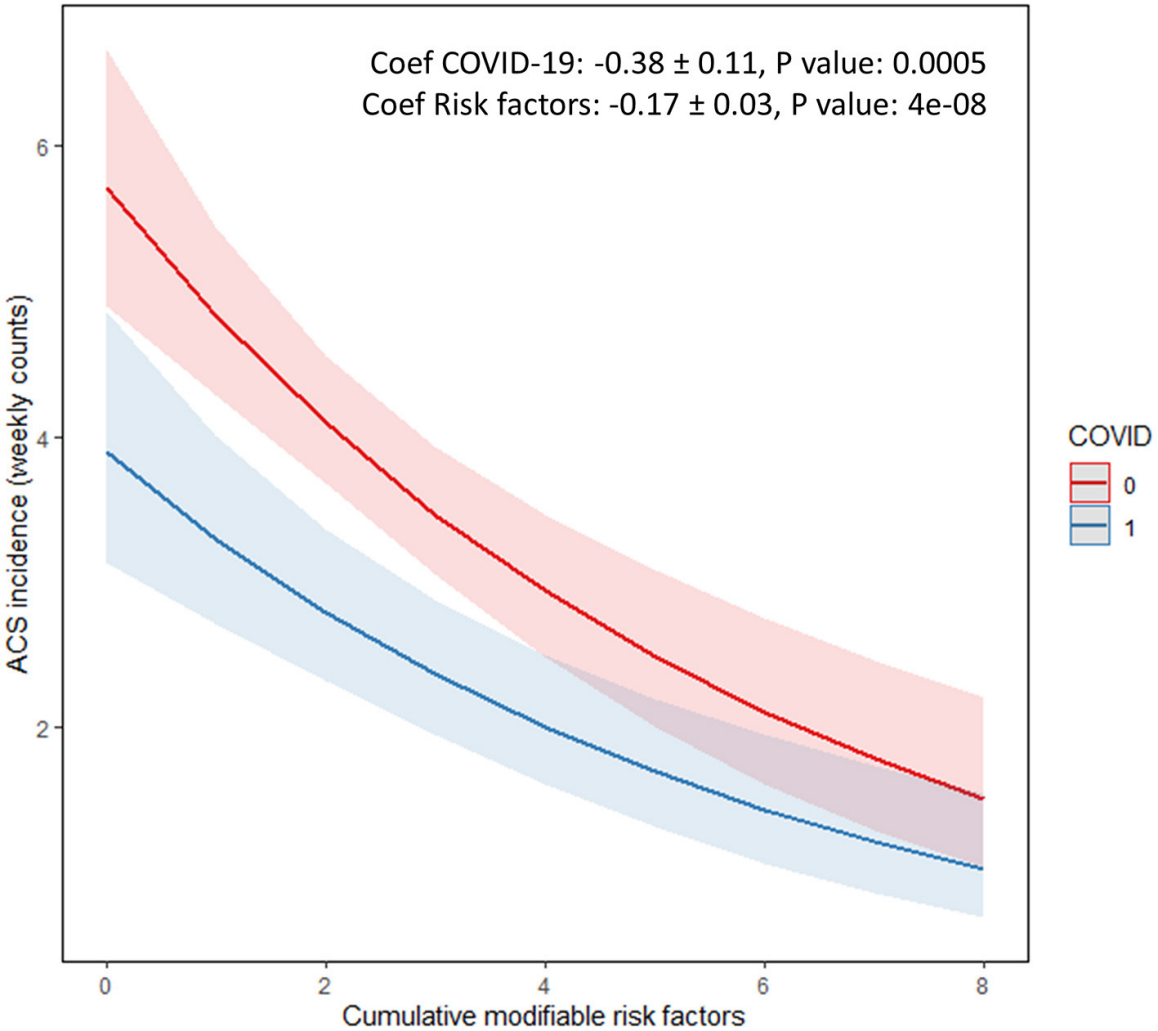
Interaction plot of Poisson regression model showing an inverse association between the reduction of the incidence of Acute Coronary Syndrome during the Covid-19 lockdown period and the number of registered patient risk factors. Cumulative modifiable risk factors (*n*) included smoking, hypertension, diabetes, dyslipidemia, renal disease, peripheral arterial disease, atrial fibrillation, familial history, and previous history of coronary artery disease. The log coefficients for the Covid-19 disease and for each added individual risk factor are noted (R package sjPlot) (0, Non-covid-19 era 2019 and 2020; 1, Covid-19 era 2020).

During the period of interest, a total of 160 ACS (34.4% STEMI) admissions were recorded in 2018, 175 (33.7% STEMI) in 2019, and 111(32.4%STEMI) in 2020. Age, gender, and all other CAD risk factors (smoking, diabetes, hypertension, hyperlipidemia), with the exception of familial history of CAD, did not differ among the admitted ACS patients (Table 1). The Median ACS incidence rate decreased from 19.0 cases per week in 2018 and 21.5 in 2019 down to 13.0 in 2020 (RR:0.66 during the lockdown; 95%CI: 0.53–0.82; *P* = 0.0002). The Median rates of coronary artery revascularizations decreased significantly from 12.5 per week in 2018 and 15.0 in 2019 down to 8.0 in 2020 (RR: 0.59 during the lockdown; 95%CI: 0.45–0.77; *P* = 0.0001). The observed decline in ACS admissions was driven by a significant reduction in patients with NSTEMI (RR: 0.68; 95%CI: 0.52–0.88; *P* = 0.0037); and correspondingly the decline in revascularizations was driven by a significant reduction in PCI procedures (RR: 0.58; 95%CI: 0.43–0.80; *P* = 0.0007; Figure 1). Conservative treatment for ACS management was largely stable across 2018, 2019, and 2020 time-intervals (RR: 0.83, 95%CI: 0.58–1.18, *P* = 0.299). Although there was a decline in the rates of STEMI across the examined time periods, this did not reach statistical significance probably due the small number of events. In the STEMI subgroup analyses, a numerical trend toward later (>24 h) admissions (*P* = 0.014) and increased patient mortality were noted (*P* = 0.03), whereas the rates of thrombolysis did not differ across the 3 time periods examined (Table 2). A numerical trend toward lower incident coronary angiograms without significant findings or ensuing treatment (thrombolysis, PCI or CABG) was also noted (RR: 0.73; 95%CI: 0.53–1.01; *P* = 0.055) in line with the rest of the aforementioned findings on the incidence of overall ACS events (Table 2). Stratified analyses of individual risk factors identified a significant reduction in ACS incidence in most cases in the absence of the known CAD risk factors (i.e., family history of coronary artery disease, smoking, diabetes, dyslipidemia, renal dysfunction, peripheral arterial disease, renal dysfunction, atrial fibrillation; *P* < 0.001 in all cases; Figure 2). There was an inverse association between the reduction in the incidence of ACS during the Covid-19 lockdown period and the burden of registered cardiovascular risk factors (Figure 3).

**Table 1:** Acute coronary syndrome (ACS), events and patient characteristics.

	**2018**	**2019**	**2020**	***P* value**
ACS cases	*n* = 160	*n* = 175	*n* = 111	Chi^2^
Male gender	127 (79.4%)	132 (75.4%)	81 (73.0%)	0.45
Age (years)	64.3 ± 13.6	65.3 ± 13.7	65.3 ± 12.3	0.49
Smoking	70 (43.8%)	74 (42.2%)	54 (48.6)	0.56
Hypertension	74 (46.3%)	95 (54.2%)	55 (49.5%)	0.34
Diabetes	34 (21.3%)	39 (22.2%)	28 (25.2%)	0.74
Dyslipidemia	48 (30.0%)	64 (36.6%)	47 (42.3%)	0.11
Renal disease	7 (4.4%)	3 (1.7%)	7 (6.3%)	0.13
Peripheral arterial disease	9 (5.6%)	15 (8.6%)	6 (5.4%)	0.46
Familial history	12 (7.5%)	32 (18.2%)	15 (13.5%)	0.01
CAD history	39 (24.4%)	36 (20.6%)	23 (20.7%)	0.66
Atrial fibrillation	12 (7.5%)	6 (3.4%)	2 (1.8%)	0.06

*ACS, Acute Coronary Syndrome; CAD, coronary artery disease*.

**Table 2:** Clinical events and outcomes in patients with ACS.

	**2018**	**2019**	**2020**	***p*-value**
Late presentation (>24 h)	2/53 (3.6%)	3/59 (5.0%)	7/29 (19.4%)	0.014
Thrombolysis	8/53 (15.1%)	7/59 (11.9%)	7/29 (24.1%)	0.33
STEMI	55/160 (34.4%)	59/175 (33.7%)	36/111 (32.4%)	0.09
NSTEMI	105/160 (65.6%)	116/175 (66.3%)	75/111 (67.6%)	0.0037
Conservative treatment^*^	70/160 (43.8%)	65/175 (37.1%)	49/111 (44.1%)	0.055
Mortality	2/160 (1.3%)	6/175 (3.4%)	8/111 (7.2%)	0.03

*P-values have been calculated with a Poisson regression model of weekly counts of events*.

* *Conservative treatment group includes all the patients who have received only medical treatment after coronary angiography, without further interventional (PCI or CABG) procedures*.

The number of all-cause deaths registered across the municipal regions (population, *n* = 411,576) was largely stable across the examined years (2018, *n* = 604; 2019, *n* = 672, and 2020, *n* = 604). All-cause mortality per 100,000 population during the examined time period, in the years 2018, 2019, and 2020 was 146.7, 163.7, and 146.7, in the years 2018, 2019, and 2020, respectively, with no differences among the examined 3 municipalities.

**Figure 4:**
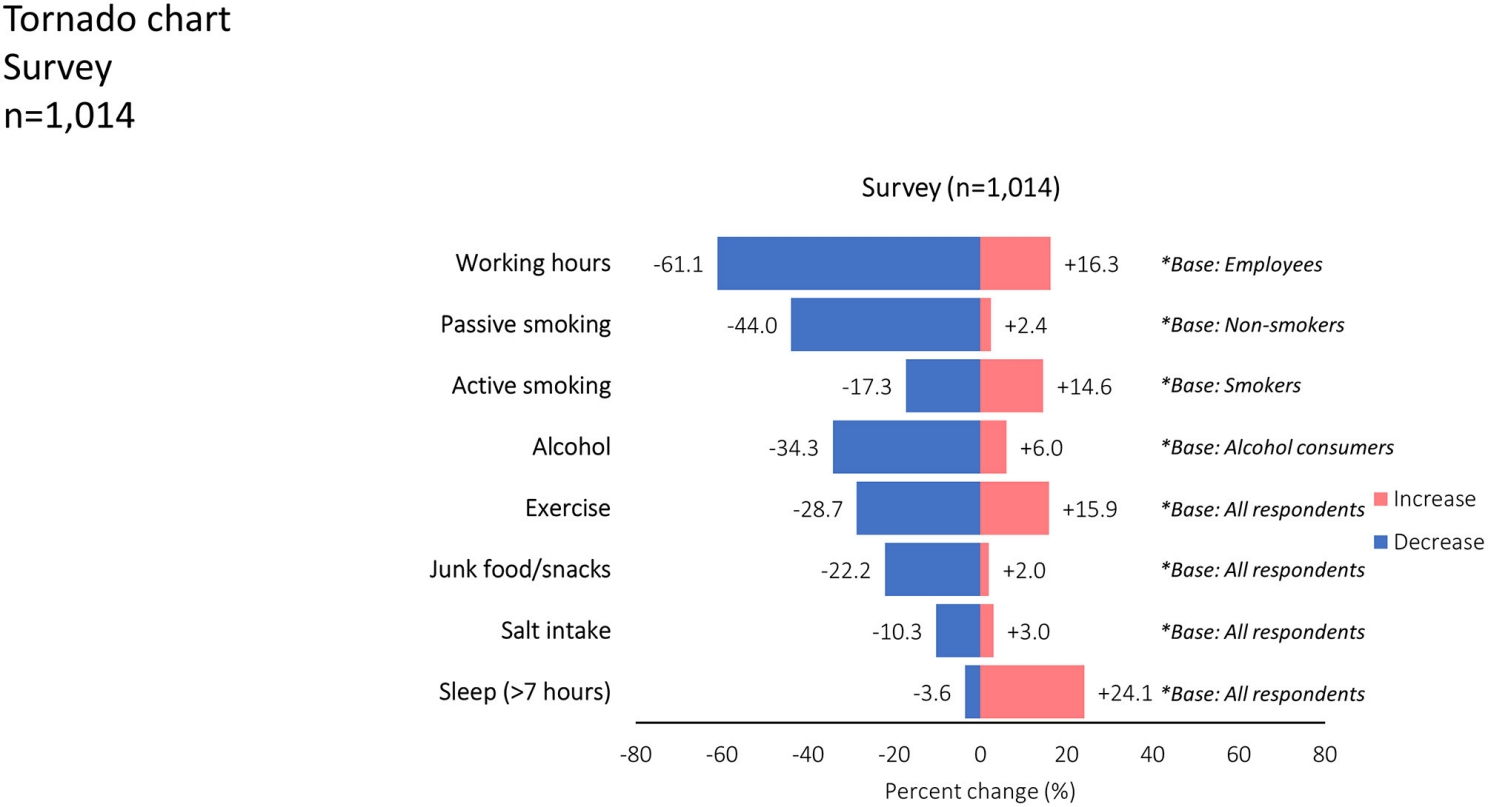
Tornado chart demonstrates the reported changes in various lifestyle variables (*P*: 0.000 in all cases except active smoking). Blue bars extending to the left-hand side refer to a decrease, whereas red bars on the right-hand side refer to an increase in the reported frequency of the lifestyle habit.

For the telephone survey, a total of 10,917 contacts were made, but 7,777 refused to answer. In 2,126 cases the interview was not conducted because the specifications were not met (e.g., age <35 years, etc.). Hence, the sample size for the telephone survey was 1,014, of whom 48.7% were women. These included 509, 302, and 203, inhabitants of the 3 municipalities. The sample was weighted by using external data on age and sex from the 2011 census in order to prevent the bias due to sample design and distribution, as well as non-response variance while making estimations. The most important results regarding lifestyle habit changes during quarantine are presented in Figure 4.

There were significant changes in most of the lifestyle variables reported with the exception of active smoking. Briefly, passive smoking (13.5% of the sample), was reduced in 44.0% of non-smokers, mainly men, and the younger (*P* < 0.001). Regular smoking (31% of the sample), was not affected during the lockdown. However, a proportion of 34.5% of occasional smokers (9% of smokers) reported a reduction in smoking during quarantine. Amongst people who reported alcohol consumption, the latter was reduced in 34.3%. This was more evident in men, younger people, the unemployed, and more highly educated individuals (*P* < 0.001). Most participants (61.1%), reported reduced working hours during the lockdown period compared to the pre-lockdown time. This was more evident in women, those aged <75 years, those with higher education status, and people with a lower family income (*P* < 0.001). The proportion of people sleeping >7 h during the lockdown was significantly greater compared to previous habits (*P* < 0.001), mainly in the younger people and those with higher income (*P* < 0.001). The increase in sleeping time and the reduction in working hours, smoking, and junk food consumption were more pronounced in participants with fewer risk factors (*P* < 0.01 for all).

There was no difference in self-reported compliance with medications for chronic diseases. People who did not exercise regularly (<3–4 times per week), reported an increase in exercise time. Among people who did not previously exercise at all, 15% reported exercising during the lockdown. Junk food, snack, and salt consumption were reduced in 25.5, 18.8, and 10.3%, respectively, in this poll. Anxiety related to the pandemic was reported by 45.1% and lack of motivation and satisfaction by the 38.4% of the participants. A proportion of 76.9% of the participants reported that if they experienced chest pain they would seek the assistance of their personal doctor.

**Figure 5:**
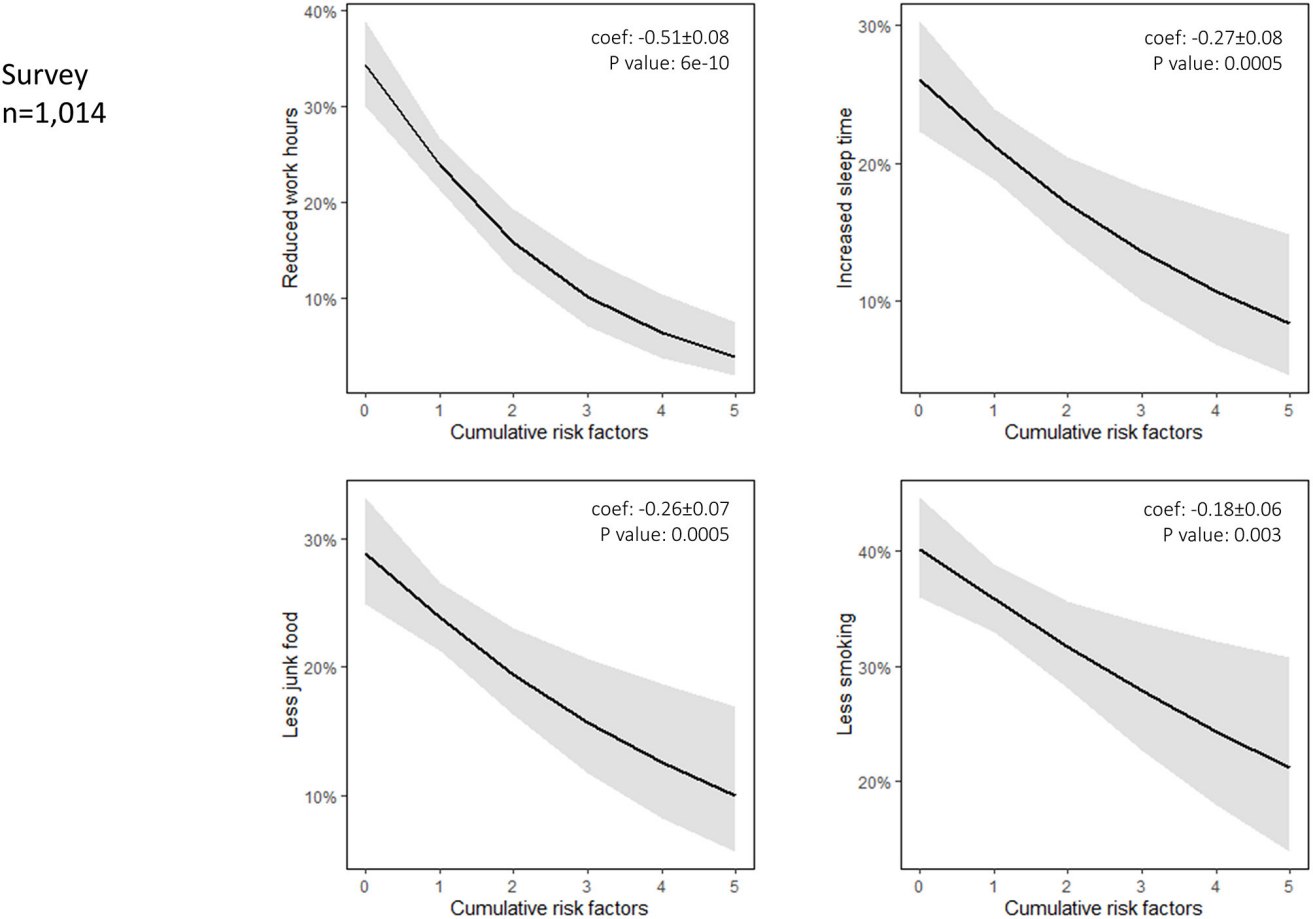
Interaction plot of a logistic regression model denotes an inverse association (negative coefficients) between the observed change in the frequency of the lifestyle habit and the number of registered patient risk factors. Cumulative modifiable risk factors (*n*) registered in the survey and introduced in the logistic regression analysis included smoking, hypertension, diabetes, dyslipidemia, and previous history of coronary artery disease. Reduced working hours, less smoking, less junk food intake, and more sleeping time were reported more frequently in patients with a lower number of modifiable cardiovascular risk factors.

An inverse association (negative coefficients) between some of the observed lifestyle changes and the number of registered patient risk factors, was noted in the survey (Figure 5). In particular, reduced work hours, less smoking, less junk food intake, and more sleeping time were reported more frequently in patients with a lower number of modifiable cardiovascular risk factors. Finally, survey participants aged <65 years reported more exercise, reduced alcohol consumption, less junk food consumption (*P* < 0.001), and more sleeping hours compared to older people (>65 years) during the lockdown period.

## Discussion

This study shows that during the lockdown period imposed by the Hellenic Republic Greek government because of the COVID-19 pandemic, there was a significant reduction in the incidence of ACS admissions in 3 spoke, and 1 hub university hospital covering 3 neighboring municipalities in southwestern Greece. This observation is in accord with worldwide experience from the USA, Europe, and other continents (1–7). The prevailing hypothetical explanations for this phenomenon include fear of contagion at the hospital, reassignment of medical services to care of COVID-19 patients, use of thrombolysis for STEMI in district hospitals, and STEMI misdiagnosis (1–7). All the above imply a false decrease in the incidence of ACS (8–10), which could potentially lead to a corresponding increase in cardiovascular and possibly all-cause morbidity and mortality (6, 11). Indeed an alarming four times higher rate of out-of-hospital cardiac arrest was reported in New York City from 30 March to 5 April 2020, associated with an eight times higher mortality compared to the same period of the previous year (12). Very recently Nef et al. reported an 11.8% increase of cardiac mortality in 2020 compared to 2019 (IRR: 1.12, 95% CI: 1.05–1.19; *p* < 0.001), in central Germany (Hesse), suggesting that patients probably presented, or were referred too late to the hospitals (6).

In our study the observed incidence of STEMI and all-cause regional mortality was largely stable and the significant cumulative decline in ACS admissions was mainly driven by a reduced incidence of NSTEMI observed primarily in patients with low cardiovascular risk. In the STEMI subgroup analyses, a numerical trend toward later (>24 h) admissions, and increased patient mortality were noted. We cannot exclude that the reduction in STEMI admissions and higher in-hospital mortality did not reach statistical significance due to the small number of events. This would be in accord with the observed higher in-hospital mortality in patients admitted for cardiac catheterization during the COVID-19 pandemic compared with 2019 (58/1,801 vs. 55/3,030, *p* = 0.002), reported by Nef et al. (6) This would support the theory of a phenomenal reduction in ACS incidence due to patients' denial to seek medical care under the fear of the pandemic. Nevertheless, an alternative scenario of “Life in a Standstill” where a real reduction of ACS incidence (mainly NSTEMI) could be related to lifestyle changes induced by lockdown measures also cannot be excluded. This hypothesis has also been proposed by others, however without any data regarding lifestyle changes during quarantine/ lockdown (13). Indeed, our survey of 1,014 citizens in our area during the lockdown revealed a significant reduction in the rate of occasional and passive smoking, working hours, and alcohol, salt and junk food consumption, along with a significant increase in sleeping hours and light to moderate exercise (in people who did not exercise before the lockdown). Many of these lifestyle habits that were changed favorably during quarantine are well-known risk factors for ACS (14–18). Hence, modification of such factors in the setting of quarantine could reduce the chance of stable coronary plaque destabilization and rupture. Most interestingly, these lifestyle changes were reported significantly more often by people with less risk factors for CAD and by relatively younger people. This parallels with the observation that the decline in ACS admissions was more pronounced in lower risk patients. It may be that the latter experienced a significant lifestyle change, thus reducing the chance for an acute plaque rupture and myocardial infarction.

It is important to stress that in contrast to other countries in Europe and the USA, Greece did not experience a severe outbreak of COVID-19 thanks to the very early institution of lockdown measures before the infection could spread in the community. Therefore, the dramatic scenes seen in hospitals of other countries such as Italy, Spain, or the USA were not observed in our country, potentially inducing less fear and hesitation in citizens to seek medical assistance if needed. Indeed, 54.9% of the participants in the survey did not report any anxiety related to the pandemic, while 61.6% did not report any depressive feelings. It should also be emphasized that the medical system in Greece is largely based on private general, and specialized medical services, largely affordable for the vast majority of citizens. Most private cardiology medical offices remained active during this period, and cardiologists were easily accessible to their private patients. Furthermore, at least 70% of the participants in our poll answered that they would seek medical care from their private physician without delay in case of chest pain, or dyspnoea. The decline in ACS admissions during the lockdown was not associated with any increase in total mortality per 100,000 population in the area covered in this study. Additionally, the rates of thrombolysis for STEMI did not increase in 2020 compared to 2018, and 2019, and our hub hospital did not discourage referral of ACS patients for catheterization. The lower proportional reduction of ACS during lockdown in patients with a higher burden of cardiovascular risk factors implies that high-risk patients with established atherosclerosis continued to suffer ACS and presented to the hospital during lockdown, whereas lower-risk patients may have actually experienced a real decrease in ACS incidence. The latter could be explained by lifestyle changes during the lockdown period, as it is an established knowledge that biological pathways, correlated with daily activities and the circadian rhythm could play an important role on the onset of ACS.

### Conclusion

A significant decline in ACS admissions during the COVID-19 lockdown was noted, affecting mainly NSTEMI patients with a lower burden of cardiovascular risk factors. This was accompanied by significant lifestyle changes. Thus, it is tempting to speculate that to some extend the latter might be associated with the observed decline in ACS admissions.

#### Limitations

The exact number of the population in the area examined was based on the last nationwide census of 2011. Since then, according to the Greek Statistical Agency (ELSTAT), there is a stable decline of the country population of 25,000–30,000. Thus, we do not expect major population changes during the years 2018–2020 in the above area. The actual causes of death in the 3 municipalities during the period of lockdown and the corresponding period in 2018, and 2019 were not available. However, there was no difference in the number of total deaths over time. Because of the study design, no direct correlation can be demonstrated between the decrease in ACS admissions and lifestyle changes by the design of the study. Thus, our results are mainly hypothesis generating, and certainly apply to the very specific scenario of countries that did not experience the devastating effects from the pandemic thanks to the early institution of preventive measures. The number of observations for both STEMI and NSTEMI were small, as the study was not nationwide; nevertheless, they were statistically significant. Furthermore, we cannot also exclude that the observed reduced incidence of ACS admissions could be partially driven by reduced rates of type II NSTEMI events that presented with negative angiograms. However, our analysis is most likely underpowered to discern between STEMI and NSTEMI subtypes. Finally, regardless of the aforementioned study limitations, in a purely observational cohort, the Covid-19 lockdown circumstances could hardly be reproduced under controlled experimental conditions (e.g., a randomized study), to confirm or refute our hypothesis and findings.

## Data Availability Statement

The raw data supporting the conclusions of this article will be made available by the authors, without undue reservation.

## Ethics Statement

The studies involving human participants were reviewed and approved by ethics committee of Patras University Hospital. Written informed consent for participation was not required for this study in accordance with the national legislation and the institutional requirements.

## Author Contributions

GT, E-EK, KK, PV, and PD contributed to conception design, analysis and interpretation, drafted, and critically revised the manuscript. PP, AM, IM, IC, FP, TD, and AK contributed to analysis and interpretation. GA contributed to analysis, drafted, and critically revised the manuscript. All authors contributed to the article and approved the submitted version.

## Conflict of Interest

The authors declare that the research was conducted in the absence of any commercial or financial relationships that could be construed as a potential conflict of interest.

## Abbreviations

ACS: acute coronary syndromes
COVID-19: coronavirus disease
USA: United States of America
DATA RC SA: Data Research and Consulting Société Anonyme
ISO: International Organization for Standardization
STEMI: ST elevation myocardial infarction
NSTEMI: Non-ST elevation myocardial infarction
PCI: Percutaneous Coronary Intervention
CABG: Coronary Artery Bypass Grafting
CATI: Computer Assisted Telephone Interviewing
GDPR: General Data Protection Regulation
ANOVA: Analysis of Variance
SPSS: Statistical Package for the Social Sciences
CAD: coronary artery disease
FMC: First Medical Contact.
